# Health Inequalities of STEMI Care Before Implementation of a New Regional Network: A Prefecture-Level Analysis of Social Determinants of Healthcare in Yunnan, China

**DOI:** 10.34172/ijhpm.2021.29

**Published:** 2021-05-11

**Authors:** Li Mei Zhang, Alan Frederick Geater, Edward B. McNeil, Yun Peng Lin, Si Chen Liu, Heng Luo, Yuan Zhang Wang, Shao Chang Wen

**Affiliations:** ^1^Department of Cardiology, People’s Hospital of Chuxiong Prefecture, Yunnan, China.; ^2^Epidemiology Unit, Faculty of Medicine, Prince of Songkla University, Hat Yai, Thailand.; ^3^Faculty of Dentistry, Prince of Songkla University, Hat Yai, Thailand.; ^4^People’s Hospital of Chuxiong Prefecture, Yunnan, China.; ^5^Executive Office, Alliance of Chuxiong Prefecture Chest Pain Centres, Yunnan, China.

**Keywords:** Health Inequality, Social Determinants, ST-elevation Myocardial Infarction, Reperfusion, Under-Developed Area, Rural-Urban

## Abstract

**Background:** As one of the most serious types of coronary heart disease, ST-elevation myocardial infarction (STEMI) faces huge challenges in the equal management and care of patients due to its life-threatening and time-critical condition. Health inequalities such as sex and age differences in STEMI care have been reported from developed countries. However, limited outcomes have been investigated and the major drivers of inequality are still unclear, especially in under-developed areas. This study aimed to explore the major drivers of health inequalities in STEMI care before implementation of a new regional network in the south-west of China.

**Methods:** Prefecture-level data of STEMI patients before the implementation of a regional network were analysed retrospectively. Drivers of inequality were identified from six social determinants of health, namely area of residence, ethnicity, sex, age, education and occupation. Outcomes of STEMI care included timely presentation, reperfusion therapy, timely reperfusion therapy, heart failure, inpatient mortality, length of hospital stay, hospital costs, and various intervals of ischaemic time.

**Results:** A total of 376 STEMI patients in the research area before implementation of the STEMI network were included. Compared with urban residents, rural patients were significantly less likely to have timely presentation (odds ratio [OR]=0.47, 95% CI: 0.28-0.80, *P*=.004) and timely reperfusion therapy (OR=0.32, 95% CI: 0.14-0.70, *P*=.005). Rural residents were less likely to present to hospital promptly than urban residents (HR=0.65, 95% CI=0.52-0.82, *P*<.001). In the first 3 hours of percutaneous coronary intervention (PCI) reperfusion delay and first 6 hours of total ischaemic time, rural patients had a significantly lower probability to receive prompt PCI (hazard ratio [HR]=0.40, 95% CI: 0.29-0.54, *P*<.001) and reperfusion therapy (HR=0.37, 95% CI: 0.25-0.56, *P*<.001) compared to urban patients.

**Conclusion:** Rural residents were a major vulnerable group before implementation of the regional STEMI network. No obvious inequalities in ethnicity, sex, age, education or occupation existed in STEMI care in Chuxiong Prefecture of China.

## Background

**Key Messages**

**Implications for policy makers**

- The influence of rural-urban inequality in ST-elevation myocardial infarction (STEMI) healthcare was much greater than that of other social determinants including ethnicity, sex, age, education and occupation. Rural-urban inequality in healthcare should be of more concern, especially for this time-critical disease.
- Obvious rural-urban inequalities still existed in an under-developed area. Improving healthcare quality in urban areas may widen the rural-urban inequalities. Policy-makers should consider the full picture of improvement in both urban and rural areas, especially for under-developed areas.
- Delays in seeking medical services and the many steps involved in reaching a capable hospital are the two major contributors to the patient and system delays, respectively. Hence, public education to heighten the awareness of rural patients regarding seeking medical service after chest pain onset and the implementation of new, effective and timely communication mechanisms between the prehospital/in-hospital stages and between the different levels of hospitals might be the most important steps in the whole process of improving healthcare for this time-critical disease.

**Implications for the public**

It is regrettable that rural patients had a significantly longer total ischaemic time than urban residents, even though the rural patients incurred the same charge and time in hospital for the care of ST-elevation myocardial infarction (STEMI) as urban patients. Rural patients’ longer ischaemic time was due in part to their late presentation for first medical contact. Compared to urban patients, rural patients had a significantly lower probability to receive percutaneous coronary intervention (PCI) at any given time during the first three hours of reperfusion time and also were less likely to receive reperfusion (either PCI or fibrinolysis) therapy at any given time during the first six hours of total ischaemic time. Hence, suspected STEMI patients (anyone suffering from sudden severe chest pain), especially those living far away from PCI hospital, should seek emergency medical service as soon as possible, instead of presenting in hospital by themselves.

Inequalities in healthcare lead to growing costs, poverty, disability, and death, and have grave social repercussions and profound economic consequences.^1^ A huge negative impact of health inequalities exists in the management of cardiovascular disease, which is the leading cause of death worldwide,^2^ especially in low- and middle-income countries.^3^ Despite many efforts to improve the management of cardiovascular disease, noticeable from the marked reduction in mortality from myocardial infarction in the last decade, there are tremendous challenges to reduce healthcare inequalities in developing countries, particularly China.^4^

 ST-elevation myocardial infarction (STEMI) is the most serious type of acute myocardial infarction as it causes full-thickness myocardial necrosis after the related coronary artery is blocked, which induces an elevation of the ST-segment of the electrocardiogram (ECG). Heart tissue begins to die within 15-30 minutes of loss of blood supply,^5^ and complete necrosis of myocardial cells at risk can take place over 3-4 hours,^6^ which means there is a finite period of time to rescue a STEMI patient from ischaemic myocardial infarction. Prompt percutaneous coronary intervention (PCI) and fibrinolysis are the two main strategies of reperfusion therapy.^7^ However, there are many steps in the whole process, from symptom onset to patient presentation, diagnosis of STEMI and emergency reperfusion.

 It has been suggested that a STEMI network that integrates an emergency system and hospitals with different levels in a local area should be implemented to offer evidence-based standard treatment.^8^ A few STEMI networks have been reported in some regions of developed countries such as the Cleveland STEMI network in the United States.^9^ However, before the recent setting up of a STEMI network in Chuxiong Prefecture in Yunnan, China, there had been no previous STEMI network in any under-developed area. Moreover, it is notable that major drivers of healthcare inequalities before implementation of the STEMI network in this low-middle income region are not well-understood. Apart from clinical indices and the health system, the choice of reperfusion strategy is influenced by many patient factors, including presentation time and the decision to receive reperfusion therapy. Hence, STEMI care may differ in relation to patient demographic characteristics, such as age, sex, ethnicity and socio-economic factors, such as place of residence, education and occupation, which may induce inequalities of healthcare.^10^

 The objective of this study was to identify the major social drivers of STEMI care inequality before the implementation of the new STEMI network in Chuxiong Prefecture, from among six social determinants of health, namely area of residence, ethnicity, sex, age, education and occupation. The current study is the first part of the research “The impact of regional STEMI network on health inequities in under-developed area of southwest China” and reflects the situation prior to implementation of the network against which the effectiveness of the network can be evaluated. Clear drivers of inequalities should be the key focus of the new STEMI network to improve the quality and to narrow the social differences of STEMI care. Moreover, the major drivers of STEMI care inequality in Chuxiong Prefecture, which is an under-developed area in south-western China, may also influence the healthcare of other diseases (such as stroke) in other similar areas.

## Methods

###  Study Setting

 Chuxiong Prefecture, which is located in north-central Yunnan province, southern China, is an economically underdeveloped area. The latest population living in rural areas was 1 789 662 and accounted for 67.3% of the total.^11^ The male:female sex ratio was 1.037:1. Chuxiong Prefecture contains one city and nine counties, and 90% of the area is mountainous. The per capita disposable income in 2019 was 36 868 yuan for urban residents and 12 015 yuan for rural residents.^11^

 The People’s Hospital of Chuxiong Prefecture, which is located in Chuxiong city, is the only hospital in the prefecture that can provide a 24-hour primary PCI service for STEMI patients. The registered system for STEMI patients was set up in the prefecture in August 2015. In January 2017, a prefecture-wide STEMI network system was initiated. The new regional network was implemented following the protocols of China’s Chest Pain Center with modification of the European Society of Cardiology standard STEMI network based on the real condition of the local health system and population. The regional STEMI network is constructed with the People’s Hospital of Chuxiong Prefecture as the core centre, local emergency medical systems as core elements, 10 non-PCI hospitals and other town hospitals and primary care units in the community and/or villages as branch elements, and involves public education and professional training. Regional information sharing and prehospital care are achieved through cloud technology and the use of the WeChat mobile application software.

###  Study Design

 Index STEMI cases were obtained from the STEMI registry database between August 2015 and the date of the implementation of the network in each county. Each case was linked to their demographic information from medical records including social determinants of health and other important clinical indices such as heart rate, blood pressure, and respiratory parameters. All regional cases diagnosed with STEMI in Chuxiong Prefecture before the implementation of STEMI network were analysed retrospectively. Patients who lived outside of Chuxiong Prefecture were excluded, Chuxiong city is defined as an urban area while the other nine counties are defined as rural areas. Han is the major ethnic group of the prefecture. The Yi people are the largest ethnic minority group, although other minorities including Hui, Bai, and Lisu are well-represented. Age group was categorised as less than 50 years, 51-60 years, 61-70 years and more than 70 years. Education was categorised as none (no formal education), primary school, secondary school and higher.

###  Assessment of STEMI Care

 Outcomes of STEMI care, namely timely presentation, reperfusion therapy, timely reperfusion therapy, heart failure, inpatient mortality, length of hospital stay, hospital cost, and various intervals of total ischaemic time were analysed.

 In real conditions of STEMI care, the time point at first diagnosis of STEMI by ECG is defined as “time zero.” The time duration from onset of symptoms to “time zero” is defined as the presentation delay, which includes patient delay (defined as the duration from onset of chest pain to first medical contact) and diagnosis delay (time from first medical contact to first diagnosis by ECG), and will guide subsequent reperfusion therapy.^8^ Timely presentation is defined as presentation with STEMI within 12 hours after onset of symptoms. If a person has had symptoms for 12 to 24 hours, then evidence suggests that the effectiveness of fibrinolysis is low. If the person has had symptoms for more than 24 hours then fibrinolysis is not recommended.^12^

 The sign of successful reperfusion is the guidewire passing the stenosis of the related coronary artery for patients who receive primary PCI, or the needle puncturing the vein for patients who receive fibrinolysis. Reperfusion delay is defined as the time from “time zero” to “wire-crossing time” (Z to W) for patients who received primary PCI, or the time from “time zero” to “needle time” (Z to N) for patients who received fibrinolytic drugs. According to the latest guideline from the European Society of Cardiology for STEMI,^8^ timely reperfusion therapy (fibrinolysis or primary PCI intervention) should be provided to patients who present within 12 hours (except for those patients with ongoing/recurrent chest pain, unstable hemodynamic status or heart arrest).

 The strategy of reperfusion therapy depends on the availability of primary PCI. If primary PCI is available within 120 minutes from “time zero,” it should be performed within 90 minutes if the place of first medical contact is a non-PCI hospital and within 60 minutes if it is a PCI hospital (timely intervention). The time from first diagnosis of STEMI in a non-PCI hospital to arrival at the catheter room is defined as the transfer time. If primary PCI is not available within 120 minutes, patients without contraindication should be given fibrinolytic drugs within 10 minutes after “time zero” (timely fibrinolysis).

 Patient delay can reflect public awareness of recognizing the common symptoms of acute myocardial infarction and seeking emergency services, while system delay, which includes diagnosis delay and reperfusion delay, is the most easily audited indicator of the quality of a STEMI care system. The length of hospital stay is the number of days from admission to discharge, and the hospital charge is the total fee for STEMI patients between their admission to and discharge from hospital.

###  Statistical Analysis

 All data analysis was performed using R. Comparison of categorical outcome variables across the levels of each determinant was initially tested using the chi-square test, while comparison of continuous outcome variables was initially tested using the Wilcoxon rank-sum test or the Kruskal-Wallis test as appropriate. Hypothetical causal relationships between social determinants of health and STEMI care outcomes were indicated using directed acyclic graphs. Kaplan-Meier curves and Cox proportional hazards regression models incorporating time-varying effects were used to estimate the total effect of each social determinant of health on each interval of ischaemic time (patient delay, diagnosis time, transfer time, reperfusion time and total ischaemic time), hospital stay and cost. The total effect of each determinant on categorical outcomes, including timely presentation, reperfusion therapy, timely reperfusion therapy, heart failure and inpatient mortality, was separately estimated using logistic regression models controlling for confounding according to the directed acyclic graph (see Figures S1 and S2 in Supplementary file 1).

## Results

###  Baseline Characteristics

 A total of 376 patients diagnosed with STEMI in Chuxiong Prefecture before the implementation of the STEMI network comprising 280 males with mean age 58.59 (SD 11.85) years and 96 females with mean age 65.76 (SD 10.43) years were analysed. Approximately 70% were from a rural area, 92% were of Han ethnicity and 60% were farmers. Half of the patients had a history of smoking and one-third were regular alcohol drinkers. About a third had a body mass index either less than 18.5 (underweight) or higher than 25.0 (overweight). The majority (95%) of patients had basic medical insurance. About 86% of patients had typical symptoms of chest pain before diagnosis but only 4.5% called for an ambulance immediately after symptoms onset. Clinical indices including heart rate, blood pressure, respiratory rate, pulse oxygen and Killip heart function, and some other related baseline characteristics of STEMI patients are shown in Table 1.

**Table 1:** Characteristics of All STEMI Patients Pre-Network

		**No. (%)**
**Social Determinants of Health**		
Area of residence	Rural	261 (69.4)
	Urban	115 (30.6)
Ethnicity	Other	32 (8.5)
	Han	344 (91.5)
Gender	Female	96 (25.5)
	Male	280 (74.5)
Occupation	Farmer	224 (59.6)
	Non-farmer	152 (40.4)
Age group (y)	<51	77 (20.5)
	51-60	97 (25.8)
	61-70	115 (30.6)
	>70	87 (23.1)
Education	None	79 (21.0)
	Primary	48 (12.8)
	Secondary	152 (40.4)
	Tertiary	97 (25.8)
**Behavioural Factors**		
Smoking	Yes	193 (51.3)
	No	183 (48.7)
Drinking	Yes	139 (37.0)
	No	237 (63.0)
BMI	Underweight	33 ( 8.8)
	Normal	239 (63.6)
	Overweight	104 (27.7)
Insurance	No	18 ( 4.8)
	Yes	358 (95.2)
Mode of transportation	Self-transport	359 (95.5)
	Ambulance	17 (4.5)
Onset area	Rural	216 (57.4)
	Urban	160 (42.6)
Onset period	Night	162 (43.1)
	Day	214 (56.9)
**Clinical Indices**		
Symptom	Atypical	51 (13.6)
	Typical	325 (86.4)
Heart rate	Bradycardia	52 (13.8)
	Normal	282 (75.0)
	Tachycardia	42 (11.2)
Blood pressure	Hypotension	100 (26.6)
	Normal	220 (58.5)
	Hypertension	56 (14.9)
Respiratory rate	Abnormal	82 (21.8)
	Normal	294 (78.2)
Pulse oxygen	<90%	8 (2.1)
	≥90%	368 (97.9)
Heart function (Killip class)	I	245 (65.2)
	II or higher	131 (34.8)
**Categorical Outcomes of STEMI Care**		
Timely presentation^a^	Yes	258 (68.6)
	No	116 (30.9)
Reperfusion	Yes	226 (60.1)
	No	150 (39.9)
Timely reperfusion	Yes	31 (8.2)
	No	345 (91.8)
Heart failure^b^	Yes	40 (10.6)
	No	334 (88.8)
Death in hospital	Yes	10 (2.7)
	No	366 (97.3)
		**Median (IQR)**
**Intervals of Ischaemic Time**		
Patient delay (h)		3.8 (1.5, 15.0)
Diagnosis time (min)		5.0 (1.0, 10.0)
Z to W time (h)		2.7 (1.5, 4.4)
Transfer time (h)		3.4 (2.2, 5.5)
Total ischaemic time (h)		7.0 (3.8, 15.8)
Hospital stay (days)		9.0 (7.0, 11.0)
Cost (10 000 yuan, RMB)		3.0 (2.7, 3.5)

Abbreviations: STEMI, ST-elevation myocardial infarction; BMI, body mass index; IQR, interquartile range. ^a^2 with missing diagnosis time. ^b^2 with unknown condition of heart failure.

 Two hundred and fifty-eight patients (68.6%) presented with STEMI within 12 hours after onset of chest pain. The overall median patient delay was 3.8 hours. The median time for diagnosis of STEMI by ECG after first medical contact was 5 minutes. Two hundred and twenty-three patients (59.3%) received prompt PCI while only 3 patients received fibrinolysis. The median time from “time zero” of diagnosis to reperfusion was 2.7 hours, and the median duration of total ischaemic time for all patients was 7 hours. Despite 60% of patients receiving reperfusion therapy, only 8.2% received it in a timely manner (see Figure S3 in Supplementary file 1). Forty patients had heart failure in hospital and the inpatient mortality rate was 2.7%.

###  Categorical Outcomes of STEMI Care

 Univariate analysis of determinants associated with categorical outcomes of STEMI care is shown in Tables 2 and 3. The total effect of each determinant on each categorical outcome, as suggested by the directed acyclic graph, is shown in Tables 4 and 5.

**Table 2:** Comparison of STEMI Care Categorical Outcomes (Timely Presentation, Reperfusion and Timely Reperfusion) Across Social Determinants of Health

**SDOH**	**Total**	**Timely Presentation** ^a^		***P*** ** Value**	**Reperfusion**		***P*** ** Value**	**Timely Reperfusion**		***P*** ** Value**
		**Yes** ** No. (%)**	**No** **No. (%)**		**Yes** ** No. (%)**	**NoNo. (%)**		**Yes** ** No. (%)**	**NoNo. (%)**	
**Total**	376	258 (68.6)	116 (30.9)		226 (60.1)	150 (39.9)		31 (8.2)	345 (91.8)	
Area of residence				**.005**			.145			**.004**
Urban	115	90 (79.6)	23 (20.4)		76 (66.1)	39 (33.9)		17 (14.8)	98 (85.2)	
Rural	261	168 (64.4)	93 (35.6)		150 (57.5)	111 (42.5)		14 (5.4)	247 (94.6)	
Ethnicity				.067			.999			.498
Han	344	241 (70.5)	101 (29.5)		207 (60.2)	137 (39.8)		30 (8.7)	314 (91.3)	
Yi or other	32	17 (53.1)	15 (46.9)		19 (59.4)	13 (40.6)		1 (3.1)	31 (96.9)	
Gender				.999			**.026**			.543
Male	280	192 (68.8)	87 (31.2)		178 (63.6)	102 (36.4)		25 (8.9)	255 (91.1)	
Female	96	66 (69.5)	29 (30.5)		48 (50.0)	48 (50.0)		6 (6.2)	90 (93.8)	
Age (y)				.905			.314			.463
<50	77	55 (71.4)	22 (28.6)		53 (68.8)	24 (31.2)		8 (10.4)	69 (89.6)	
51-60	97	68 (70.1)	29 (29.9)		56 (57.7)	41 (42.3)		10 (10.3)	87 (89.7)	
61-70	115	76 (66.7)	38 (33.3)		69 (60.0)	46 (40.0)		9 (7.8)	106 (92.2)	
>70	87	59 (68.6)	27 (31.4)		48 (55.2)	39 (44.8)		4 (4.6)	83 (95.4)	
Education				.037			**.019**			.091
None	79	63 (80.8)	15 (19.2)		52 (65.8)	27 (34.2)		11 (13.9)	68 (86.1)	
Primary	48	28 (58.3)	20 (41.7)		20 (41.7)	28 (58.3)		1 (2.1)	47 (97.9)	
Secondary	152	99 (65.6)	52 (34.4)		89 (58.6)	63 (41.4)		10 (6.6)	142 (93.4)	
Higher	97	68 (70.1)	29 (29.9)		65 (67.0)	32 (33.0)		9 (9.3)	88 (90.7)	
Occupation				.058			.270			.129
Other	152	113 (74.8)	38 (25.2)		97 (63.8)	55 (36.2)		17 (11.2)	135 (88.8)	
Farmer	224	145 (65.0)	78 (35.0)		129 (57.6)	95 (42.4)		14 (6.2)	210 (93.8)	

Abbreviations: STEMI, ST-elevation myocardial infarction; SDOH: Social determinants of health. ^a^2 with missing diagnosis time. *P* values from chi-square or Fisher exact test.

**Table 3:** Comparison of STEMI Care Categorical Outcomes (Heart Failure and Death in Hospital) Across Social Determinants of Health

**SDOH**	**Total**	**Heart Failure** ^a^		***P*** ** Value**	**Death in Hospital**		***P*** ** Value**
		**Yes** ** No. (%)**	**No** **No. (%)**		**Yes** **No. (%)**	**No** **No. (%)**	
**Total**	376	40 (10.6)	334 (88.8)		10 (2.7)	366 (97.3)	
Area of residence				.310			.502
Urban	115	9 (7.8)	106 (92.2)		4 (3.5)	111 (96.5)	
Rural	261	31 (12.0)	228 (88.0)		6 (2.3)	255 (97.7)	
Ethnicity				**.035**			.999
Han	344	40 (11.7)	302 (88.3)		10 (2.9)	334 (97.1)	
Other	32	0 (0.0)	32 (100.0)		0 (0.0)	32 (100.0)	
Sex				.999			.999
Male	280	30 (10.8)	248 (89.2)		8 (2.9)	272 (97.1)	
Female	96	10 (10.4)	86 (89.6)		2 (2.1)	94 (97.9)	
Age (y)				.127			.645
<50	77	3 (3.9)	73 (96.1)		2 (2.6)	75 (97.4)	
51-60	97	10 (10.4)	86 (89.6)		2 (2.1)	95 (97.9)	
61-70	115	17 (14.8)	98 (85.2)		2 (1.7)	113 (98.3)	
>70	87	10 (11.5)	77 (88.5)		4 (4.6)	83 (95.4)	
Education				.446			**.022**
None	79	6 (7.6)	73 (92.4)		6 (7.6)	73 (92.4)	
Primary	48	6 (12.5)	42 (87.5)		1 (2.1)	47 (97.9)	
Secondary	152	14 (9.3)	136 (90.7)		1 (0.7)	151 (99.3)	
Tertiary	97	14 (14.4)	83 (85.6)		2 (2.1)	95 (97.9)	
Occupation				.797			.212
Other	152	15 (9.9)	137 (90.1)		6 (3.9)	146 (96.1)	
Farmer	224	25 (11.3)	197 (88.7)		4 (1.8)	220 (98.2)	

Abbreviations: STEMI, ST-elevation myocardial infarction; SDOH, Social determinants of health. ^a^2 with unknown condition of heart failure. *P* values from chi-square or Fisher exact test.

**Table 4:** Logistic Regression of STEMI Care Categorical Outcomes (Timely Presentation, Reperfusion and Timely Reperfusion) Across SDOH

**SDOH**	**Timely Presentation** ^a^			**Reperfusion** ^b^			**Timely Reperfusion†**			**Minimal Aadjustment Set**
	**Crude OR (95%CI)**	**Adjusted OR (95% CI)**	***P***	**Crude OR (95% CI)**	**Adjusted OR (95% CI)**	***P***	**Crude OR (95% CI)**	**Adjusted OR (95% CI)**	***P***	
Area (Ref. Urban)			**.004**			.179			**.005**	Age, ethnicity, sex.
Rural	0.46 (0.27, 0.78)	0.47 (0.28, 0.80)		0.69 (0.44, 1.10)	0.73 (0.46, 1.16)		0.34 (0.15, 0.73)	0.32 (0.14, 0.70)		
Ethnicity (Ref. Han)			**.049**			.930			.231	No adjustment set is necessary.
Yi or other	0.47 (0.23, 0.99)	0.47 (0.23, 0.99)		0.97 (0.46, 2.02)	0.97 (0.46, 2.02)		0.34 (0.04, 2.64)	0.34 (0.04, 2.64)		
Sex (Ref. Male)			.905			**.020**			.777	No adjustment set is necessary.
Female	1.03 (0.62, 1.71)	1.03 (0.62, 1.71)		0.57 (0.36, 0.92)	0.57 (0.36, 0.92)		0.87 (0.34, 2.27)	0.87 (0.34, 2.27)		
Age (Ref. ≤50 years)			.905			.376			.514	No adjustment set is necessary.
51-60	0.94 (0.49, 1.81)	0.94 (0.49, 1.81)		0.63 (0.34, 1.17)	0.63 (0.34, 1.17)		1.17 (0.42, 3.24)	1.17 (0.42, 3.24)		
61-70	0.80 (0.43, 1.50)	0.80 (0.43, 1.50)		0.70 (0.38, 1.27)	0.70 (0.38, 1.27)		0.79 (0.28, 2.21)	0.79 (0.28, 2.21)		
>70	0.87 (0.45, 1.71)	0.87 (0.45, 1.71)		0.59 (0.31, 1.12)	0.59 (0.31, 1.12)		0.49 (0.14, 1.74)	0.49 (0.14, 1.74)		
Education (Ref. Higher)			**.050**			.269			.564	Area, ethnicity, sex, age.
None	1.79 (0.88, 3.65)	1.74 (0.84, 3.59)		0.95 (0.51, 1.78)	0.92 (0.49, 1.74)		1.66 (0.63,4.40)	1.46 (0.53,4.01)		
Primary	0.60 (0.29, 1.23)	0.51 (0.22, 1.17)		0.35 (0.17, 0.72)	0.45 (0.20, 1.02)		0.31 (0.04,2.61)	0.39 (0.04,3.88)		
Secondary	0.81 (0.47, 1.41)	0.80 (0.44, 1.44)		0.70 (0.41, 1.18)	0.76 (0.43, 1.34)		0.75 (0.28,1.96)	0.83 (0.29,2.36)		
Occupation (Ref. Other)			.569			.942			.732	Area, ethnicity, sex, age, education.
Farmer	0.63 (0.39, 0.99)	0.86 (0.5, 1.46)		0.77 (0.50,1.18)	0.98 (0.6,1.61)		0.55 (0.26,1.18)	0.85 (0.33,2.17)		

Abbreviations: STEMI, ST-elevation myocardial infarction; SDOH, Social determinants of health; OR, odds ratio. ^a^ Excluding patients with unknown diagnosis time. ^b^Excluding patients who did not receive emergency reperfusion.

**Table 5:** Logistic Regression of STEMI Care Categorical Outcomes (Heart Failure and Death in Hospital) across Social Determinants of Health

**SDOH**	**Heart Failure**			**Death In Hospital**			**Minimal Adjustment Set**
	**Crude OR (95%CI)**	**Adjusted OR (95% CI)**	***P***	**Crude OR (95%CI)**	**Adjusted OR (95% CI)**	***P***	
Area (Ref. Urban)			.210			.618	Age, ethnicity, sex.
Rural	1.60 (0.74, 3.48)	1.63 (0.74, 3.60)		0.65 (0.18, 2.36)	0.72 (0.20, 2.62)		
Ethnicity (Ref. Han)			.008			.090	No adjustment set is necessary.
Yi or other	0.03 (0.00, 0.41)	0.03 (0.00, 0.41)		0.06 (0.00, 1.55)	0.06 (0.00, 1.55)		
Sex (Ref. Male)			.918			.676	No adjustment set is necessary.
Female	0.96 (0.45, 2.05)	0.96 (0.45, 2.05)		0.72 (0.15, 3.47)	0.72 (0.15, 3.47)		
Age (Ref. ≤50 years)			.088			.656	No adjustment set is necessary.
51-60 years	2.83 (0.75,10.67)	2.83 (0.75, 10.67)		0.79 (0.11, 5.74)	0.79 (0.11, 5.74)		
61-70 years	4.22 (1.19,14.94)	4.22 (1.19, 14.94)		0.66 (0.09, 4.81)	0.66 (0.09, 4.81)		
>70 years	3.16 (0.84,11.94)	3.16 (0.84, 11.94)		1.81 (0.32,10.15)	1.81 (0.32,10.15)		
Education(Ref. Higher)			.380			.038	Area, ethnicity, sex, age.
None	0.49 (0.18, 1.33)	0.53 (0.19, 1 49)		3.90 (0.77, 19.91)	3.87 (0.74,20.19)		
Primary	0.85 (0.30, 2.36)	0.62 (0.19, 2.01)		1.01 (0.09, 11.43)	0.70 (0.05,11.01)		
Secondary	0.61 (0.28, 1.34)	0.48 (0.21, 1.13)		0.31 (0.03, 3.52)	0.29 (0.02, 3.50)		
Occupation(Ref. Other)			.691			.851	Area, ethnicity, sex, age, education.
Farmer	1.16 (0.59, 2.28)	1.17 (0.54, 2.55)		0.44 (0.12, 1.59)	1.16 (0.26, 5.23)		

Abbreviations: STEMI, ST-elevation myocardial infarction; SDOH, Social determinants of health; OR, odds ratio.

 Compared with urban residents, patients from rural areas were significantly less likely to have timely presentation (odds ratio [OR] = 0.47, 95% CI: 0.28, 0.80, *P* = .004) and timely reperfusion therapy (OR = 0.32, 95% CI: 0.14, 0.70, *P* = .005). After adjusting for age, ethnicity and gender, there was no significant rural-urban inequality in reperfusion rate, heart failure or inpatient mortality.

 Compared with Han people, ethnic minorities were less likely to have timely presentation (OR = 0.47, 95% CI: 0.23, 0.99, *P* = .049). Ethnicity inequality was not found in reperfusion therapy, timely reperfusion therapy or in-patient mortality of STEMI care. However, ethnic minorities were less likely to have heart failure (OR = 0.03, 95% CI: 0.00, 0.41, *P* = .008) compared with Han.

 There were no sex differences in any other outcomes except that females were significantly less likely to receive reperfusion therapy (OR = 0.57, 95% CI: 0.36, 0.92, *P* = .020).

 Disparity of timely presentation, reperfusion therapy, timely reperfusion and mortality was not found among age groups. Those aged between 61 and 70 years were more likely to have heart failure (OR = 4.22, 95% CI: 1.19, 14.94, *P* = .026) compared with younger patients (≤50 years).

 Except for death in hospital, educational disparity in any other categorical outcomes of STEMI care was not found after adjusting for area of residence, sex, age and ethnicity. The *P* value of.038 for death in hospital indicates evidence for an education effect in which death in hospital is more likely among patients with no formal education.

 After adjusting for area of residence, gender, age, ethnicity and education, inequality in timely presentation rate, reperfusion rate, timely reperfusion rate, heart failure and mortality was not found between farmers and non-farmers.

###  Continuous Outcomes of STEMI Care Processing

 Kaplan-Meier analysis revealed area of residence, education and occupation to be significant factors of more than one interval of ischaemic time (see Figures S4.1 to S4.6 in Supplementary file 1). Numerical summaries for each outcome across determinants are shown in Tables S1, S2 and S3 in Supplementary file 2.

 Compared with urban residents, rural patients had a lower probability to seek medical service early; the median patient delay for rural residents being 5.0 hours while that for urban patients was 2.5 hours (*P* < .001). After diagnosis with STEMI by ECG, rural cases also showed a lower probability to receive prompt PCI. The median reperfusion time for rural residents was 3.2 hours, which was twice the median “Z to W” time of urban patients (1.5 hours) (*P* < .001). A marked rural-urban inequality existed in the total ischemic time. From onset of chest pain to receiving reperfusion therapy, the median time for patients from rural and urban areas were significantly different (8.2 vs. 3.8 hours, respectively, *P* = .003).

 Except for patient delay, there were no significant differences in the distributions of other intervals of ischaemic time between the Han (3.6 hours) and Yi or other ethnic group (9.0 hours, *P* = .007). No disparity of any interval of ischaemic time was evident across sex or age group. Education showed some differences in reperfusion time (uneducated 1.6 hours, primary 3.0 hours, secondary 3.1 hours, higher 2.6 hours, *P* = .030) and total ischaemic time (uneducated 3.9 hours, primary 6.3 hours, secondary 9.0 hours, higher 7.8 hours, *P* < .001). The uneducated group had the shortest reperfusion time and ischaemic time. Regarding occupation, the median “Z to W” time and total ischaemic time for farmers were significantly longer than for non-farmers (3.2 vs. 1.8 hours, *P* < .001, and 8.99 vs. 4.38 hours, *P* < .001).

 To adjust for potential confounders and assess the total effect of each social determinant on STEMI care outcomes, variables in the minimal adjustment sets according to the direct acyclic graphs were included in the Cox regression models. Tables S4, S5 and S6 in Supplementary file 2 summarize the results for each continuous outcome of STEMI care in terms of hazard ratios (HRs).

 After adjusting for age, ethnicity and sex, there were no significant rural-urban inequalities in diagnosis time, transfer time, hospital stay or hospital cost. However, compared with urban residents, rural residents were significantly less likely at any given time to present in hospital following onset of symptoms (HR = 0.65, [0.52, 0.82], *P* < .001), to receive PCI following STEMI diagnosis by ECG (HR = 0.42, [0.31, 0.57], *P* < .001), and to have ended their total time of ischaemia (HR = 0.66, [0.50, 0.88], *P* = .005).

 Compared with Han people, those belonging to ethnic minority groups were less likely to present to hospital promptly (HR = 0.60, [0.41, 0.87], *P* = .004). However, no ethnic inequality was found in other intervals of ischaemic time, hospital stay or cost. There were also no sex differences in any interval of ischaemic time.

 Except for duration of hospital stay there was no significant disparity of any other outcome among the four age groups. With increasing age, patients were more likely to have longer hospital stay (HR_trend_ = 0.89, [0.81, 0.98], *P*_trend_ = 0.022)

 After adjusting for area of residence, age, ethnicity and sex, educational disparity was seen only in total ischaemic time (*P* = .008). Patients with no formal education tended to have a shorter total ischaemic time (HR = 1.80, [1.23, 2.64], *P* = .003) compared to patients who received a tertiary level of education.

 Despite the fact that there was no significant difference in patient delay or diagnosis time between farmers and non-farmers, farmers had a longer PCI reperfusion time (HR = 0.55, [0.39, 0.78], *P* < .001), transfer time (HR = 0.62, [0.40, 0.98], *P* = .045) and total ischaemic time (HR = 0.64, [0.45, 0.91], *P* = .013). However, there were no significant differences in length of hospital stay or hospital charge between farmers and non-farmers.

 Tests of the proportional hazards assumption for the models of association of “Z to W” time and total ischaemic time with urban/rural resendence yielded very small *P* values indicating a non-constant HR over time. Hence, time-varying effect models were used for further analysis of “Z to W” time and total ischaemic time (see Table S7 in Supplementary file 2). Compared to urban patients, rural patients had a significantly lower probability to receive PCI at any given time during the first 3 hours of “Z to W” time (HR = 0.29, 95% CI = 0.20-0.42, *P* < .001), and also were less likely to receive reperfusion (either PCI or fibrinolysis) therapy at any given time during the first 6 hours of total ischaemic time (HR = 0.37, 95% CI = 0.25-0.56, *P* < .001). After three hours of PCI reperfusion time, and six hours of total ischaemic time, there were no significant differences between rural and urban residents. Thus, inequalities in “Z to W” time and total ischaemic time existed for residential area but only within the first 3 and 6 hours, respectively.

## Discussion

 Area of residence was found to be the major driver of STEMI care inequality before implementation of the new network in Chuxiong Prefecture, which dramatically influenced both patient presentation time and “Z to W” time. Residing in a rural area was associated with (1) longer patient presentation time and (2) longer Z to W time. Other social determinants including ethnicity, sex, age, education and occupation influenced some of the outcome variables such as time intervals.

 The purpose of identifying vulnerabilities in this context is not to label, disempower or stigmatize affected individuals or groups but to emphasize ethical obligations in healthcare and health services to protect disadvantaged groups from harm and respond to both the intrinsic and situational needs of those who are vulnerable.^13^ According to the literature, health inequities in STEMI care exist worldwide – huge sex imbalance^14^ or age differences^15^ have been reported in several studies.^16^ However, many previous studies have mentioned only two or three social determinants of health. The total effects of other social determinants have not been studied carefully or deeply.

###  Effect of Residential Area

 Rural residents were found to be a vulnerable group in STEMI care in this study. Rural patients were significantly less likely to have timely presentation and timely reperfusion therapy than urban patients. Moreover, in the first 3 hours of PCI reperfusion delay and first 6 hours of total ischaemic time, rural patients had a significantly lower probability of receiving prompt PCI and reperfusion therapy compared to urban patients. This result is consistent with other studies both in other developed/developing countries and in China.

 The largest study related to rural-urban inequality in STEMI care comes from Canada.^17^ In that study non-metropolitan patients had a longer patient delay (95 minutes vs 68 minutes; *P* < .001), a lower rate of receiving primary PCI (22.9% vs 57.4%; *P* < .001), and a significantly longer primary PCI delay time (time from first medical contact to receiving primary PCI) compared with metropolitan patients (median time 198.5 minutes vs 81 minutes, *P* < .001). Two studies in Scotland found that remote patients were significantly less likely to receive optimal reperfusion therapy than central patients (65% vs 41% )^18^ and the median time from first medical contact to initiation of thrombolysis for rural patients was 125 (range 104 to 140) minutes^19^ while that for patients from urban areas was 80 (range 78 to 93) minutes. Rural-urban inequalities in STEMI care was also found in two studies from India. One study^20^ reported that rural residence was an independent predictor of delayed presentation with an OR of 2.35 (95% CI 1.60-3.46). The other Indian study^21^ reported that prehospital delay was significantly longer among patients residing in rural areas. A very large Chinese study,^4^ involving 438 million person-years of follow up, reported that standardized mortality for rural males was significantly higher than that for urban males by 6.4% per year (95% CI: 3-10%).

 China is the most populated country in the world, with a dramatic trend towards urbanization. In 2000, 64% of China’s population was rural; by 2010, approximately half the population was living in urban areas. In 2019, the proportion of Chinese urban residents was nearly 60%. It is not unexpected that rural residents are a disadvantaged group with regard to STEMI care. As the core element of social determinants of health in STEMI care, place of residence not only influences patients’ awareness at the individual level but also markedly drives the diversity of accessibility to STEMI care. Many Chuxiong residents, especially those living in rural and mountainous areas, have weak awareness of how to recognize the symptoms of acute myocardial infarction and how to seek emergency care if symptoms occur.^22^ It is therefore difficult for most rural residents to present to a medical centre for timely therapy. Most patients suffering from severe chest pain do not seek medical contact immediately.

 On the other hand, the problems in STEMI treatment in underdeveloped areas may also include a lack of effective “green channels” (ie, priority to be diagnosed and treated) in primary PCI-capable hospitals and inadequate diagnostic capacities of community hospitals. Very few health personnel who work in a town hospital or primary care unit can provide prompt intervention therapy. Moreover, a lack of effective and timely communication mechanisms between the prehospital/in-hospital stages and between the different levels of hospitals is a major cause of the delay. Emergency Medical Service ambulances usually take chest pain patients to the nearest hospital regardless of the hospital’s treatment capacity, which frequently results in a second referral for many STEMI patients. Suspected STEMI patients might be transported from a primary care unit to a town hospital, and subsequently be transported from the town hospital to a county hospital, finally arriving at the prefecture-level hospital which provides primary PCI. Precious time is wasted due to the “step-by-step transportation” from non-PCI hospitals to the catheter room, especially in the vital first 3 hours.

 Based on current research, patient delay and the first 3 hours of reperfusion therapy are the two focuses of rural-urban inequalities in STEMI care, while there are no obvious rural-urban inequalities in diagnosis time, reperfusion rate, length of hospital stay, or hospital charge. The first 3 hours is called the “golden time” for STEMI patients due to the extent of ischaemic myocardium increasing with ischaemic time. The benefit of reperfusion therapy is time-dependent from the first moment of occlusion. Reimer et al^23^ described the wavefront phenomenon of myocardial infarction propagation after circumflex artery ligation in dogs, in which the area of necrosis was compared with the occluded artery at different times. Fully 55% of the myocardium at risk was salvageable at 40 minutes of occlusion, then the salvageable area decreased to 33% by 3 hours of occlusion, and by 6 hours, only 16% remained salvageable.

 It is regrettable that rural patients had a significantly longer total ischemic time than urban residents, even though the rural patients incurred the same charge and time in hospital for STEMI care as urban patients. Clearly, rural patients’ longer ischemic time was due to their late presentation, which includes seeking medical care, the steps involved in reaching a PCI hospital, and the ability to make the diagnosis of STEMI. Most of all, delays in seeking medical services and the many steps involved in reaching a PCI-capable hospital are the two major contributors to the delay. Hence, public education to heighten the awareness of rural patients regarding seeking medical services after chest pain onset and the implementation of a new, effective, and timely communication mechanisms between the prehospital/in-hospital stages and between the different levels of hospitals are likely to be the most important steps in the whole process of STEMI care. Furthermore, although the distance from a PCI center and rural areas cannot readily be decreased in the short term, enhancing the accessibility to reperfusion therapy (such as fibrinolysis) in rural areas is the most feasible method to reduce the steps in receiving reperfusion therapy and finally shorten the reperfusion time and system delay.

###  Effect of Ethnicity

 Chuxiong is one of two Yi autonomous prefectures in China (the other is Liangshan Prefecture in Sichuan province). Among the permanent residents, the Yi minority population accounts for around 29.3% of the total population, while other minority groups, including Lisu, Miao, Dai, Hui, and Bai, make up around 7.1%.^11^ Ethnic inequality in reperfusion therapy, timely reperfusion therapy, or in-patient mortality of STEMI care was not seen in this study, and, except for patient delay, ethnic inequality was not seen in any other intervals of ischaemic time, hospital stay, or hospital charge. Yi and other ethnic minorities were less likely to present to hospital promptly (OR = 0.47) compared with Han. However, unexpectedly, ethnic minorities were less likely to have heart failure (OR = 0.03).

 The ethnic differences of STEMI care in having heart failure may be due to the very low admission rate of Yi and other minorities, which may mean that those ethnic minority groups who have severe symptoms are not seeking medical services. Only 8.5% of STEMI patients were from a minority ethnic group while the proportion in Chuxiong is almost 30%. Ethnic minorities usually live in mountainous areas and have poor awareness of seeking medical services. Although the incidence of STEMI in ethnic minorities is unknown, it is unlikely to be lower than that among Han, as Yi people usually like to eat salty food and drink alcohol – behaviours that are clear risk factors of STEMI and other coronary heart diseases. It has been reported that some traditional ethnic minorities prefer to stay at home or refuse interventional procedures if they suffer from a serious disease or are close to dying. In a study from New Zealand,^24^ Māoris and Pacific Islanders, two minority groups, had intervention rates for PCI of 22% to 32% lower than the expected rate of STEMI hospitalization.

###  Effect of Sex

 Females were significantly less likely to receive reperfusion therapy in the current study. This result is consistent with a US study,^25^ conducted on 13 744 patients, which found that women received significantly less frequent angioplasty with stent and received reperfusion treatment less often than men. The lower reperfusion rate may be due to the unadventurous personality of women. A study from Korea,^26^ conducted on 350 patients with STEMI, found that, compared to males, females had a higher proportion of ≥60-minute decision time (33.9% vs 23.1%) and ≥120-minute arrival time (60.9% vs 52.1%).

 However, no sex disparity of any other categorical outcomes or of any interval of ischaemic time was evident in the current study. This contrasts with several studies reporting sex differences in STEMI care from China and other developed countries. A Chinese study^27^ on 1429 STEMI patients reported that women were more likely to delay seeking timely medical care. A Vienna STEMI registry study^28^ reported that females were more likely to have delays in presentation (OR = 1.35), and a study in Italy^29^ on 6022 STEMI patients found that the overall time from symptom onset to hospital presentation was longer for women compared to men (median: 270 minutes versus 240 minutes). The US study^25^ mentioned previously also reported that women were more likely to die in-hospital than men.

 Differences in the personality of women from different areas and the attitude of caregivers might be the reason for the conflicting results between the current and other studies.

###  Effect of Age

 Age had a direct influence on the health condition of STEMI patients. Length of hospital stay increased with increasing age. Those aged between 61 and 70 years were more likely to have heart failure (OR = 4.22) compared with younger patients (aged ≤50 years). In the current study, disparity among age groups was not found in timely presentation, reperfusion therapy, timely reperfusion or mortality, or any intervals of ischaemic time and hospital charge. In contrast, a Japanese study^30^ reported results of 2428 STEMI patients and found that age >75 years was a predictor for delay in door-to-balloon time. In a Chinese study^27^ on 1429 STEMI patients, elderly men (aged ≥65 years) and women (aged ≥75 years) were more likely to delay seeking timely medical care. Arguably, elderly age is a predictor for any type of delay. Patient delay and decision time to intervention therapy for elderly patients are not only related to old people themselves but also depend on their caregivers.

###  Effect of Education

 There were no significant differences in patient delay among education groups in univariate or multivariate analysis. The educational difference of reperfusion time in the Kaplan-Meier curve became non-significant in the adjusted Cox model. After adjusting for area of residence, age, ethnicity and sex, educational disparity was seen only in total ischaemic time. Patients with no formal education had a shorter total ischaemic time (HR = 1.80, [1.23, 2.64], *P* = .003) compared to patients who received a tertiary level of education, while there was no significant disparity for those with primary or secondary education. On the contrary, a study^31^ conducted on 1088 Chinese STEMI in-patients reported that higher education was associated with shorter pre-hospital delays (OR = 0.69, *P* = .006).

 Clinicians will not be surprised to discover that patients with no formal education have a shorter ischemic time due to their high-level of compliance. The most difficult part to deal with is the “half-understanding” group. Compared with non-educated patients, those with a tertiary education tend to be more inquisitive and want or expect more information when they are asked the question “Do you agree to have PCI or not?” Instead of trusting the doctor completely, most patients with a higher education commonly call their friends or other social relations before making a decision, while the uneducated patients will promptly agree to have the interventional operation. The tendency of the higher educated to have longer reperfusion time (“Z” to “W” time) is shown in Table 5, although the differences were not significant.

###  Effect of Occupation 

 After adjusting for area of residence, sex, age, ethnicity and education, no inequalities between farmers and non-farmers in timely presentation rate, reperfusion rate, timely reperfusion rate, heart failure or mortality were found, nor were there any inequalities in length of hospital stay or cost. Despite the fact that there was no significant difference in patient delay or diagnosis time between farmers and non-farmers, farmers had a longer PCI reperfusion time (HR = 0.55, [0.39, 0.78], *P* < .001), transfer time (HR = 0.62, [0.40, 0.98], *P* = .045) and total ischaemic time (HR = 0.64, [0.45, 0.91], *P* = .013).

 The effects of occupation on STEMI care have not been mentioned in any previous reports. Several studies referred to differences in oral health^32^ among occupational social classes. However, unlike oral health, STEMI is time-critical, may be fatal, and has long-term implications for the quality of life. STEMI care of a patient is not only an individual choice but also a social problem, especially in underdeveloped areas. As the most common occupation group in China, farmers with their own unique features were usually thought to be a disadvantaged group in the past. But in recent decades, the Chinese government has been paying more attention to farmers and providing some convenient and favourable measures to improve their lives. It is satisfying that farmers and non-farmers were found to have the same timely presentation rate, reperfusion rate, and timely reperfusion rate. The small occupational difference in reperfusion time and transfer time between farmers and non-farmers is expected to be reduced in the near future.

###  Limitation and Strength 

 The limitation of the study includes at least the following three issues: (1) The definition of rural and urban is not precise. The exact geographic boundary of rural and urban cannot be found in public literature or government reports. (2) A few STEMI cases might be out of registry. The study did not have access to those patients who might contact medical service in private hospital or refuse to be transferred to chest pain center or depart hospital before admission. (3) It was estimated that a portion of deaths from ischemic heart disease happen before medical contacts. However, this research could access only the STEMI patients who made medical contacts, as the diagnosis of STEMI should be made by ECG.

 The available data from the start of registration and before implementation of the network included only these 376 patients that met the inclusion criteria. It was not possible to choose a larger sample size. However, we did find evidence for a number of inequalities in the care of STEMI patients indicating that the sample did indeed have adequate power to detect those predictors of inequality. The largest study related to rural-urban inequality in STEMI care comes from Canada,^17^ in which non-metropolitan patients had a lower rate of receiving primary PCI (22.9% vs 57.4%; *P* < .001). With our rural:urban ratio of around 2.3:1 such a difference could be detected (α = 0.05) with a power of 90% with only 112 participants.

 Until now, no study on regional STEMI networks has been reported in under-developed areas of the world. Little information is available about STEMI care in rural areas of China. Almost all studies mentioned only two or three indices of the entire process of STEMI reperfusion care, and most addressed differences which were caused by only one or two factors such as age and/or sex while mentioning the diversities of STEMI management. In order to evaluate the overall effectiveness of the regional STEMI network on healthcare inequities, it is necessary to identify the major drivers of inequalities in STEMI care before the implementation of network.

## Conclusion

 Rural residents were a major vulnerable group before implementation of the regional STEMI network. No obvious inequalities in ethnicity, sex, age, education or occupation in STEMI care were identified in Chuxiong Prefecture of China. The rural-urban inequality is an important focus for evaluation of improvements provided by the newly implemented STEMI network and the effect they may have on the quality of STEMI care in this under-developed area of China.

## Acknowledgements

 The authors thank all the staff of the Chest Pain Center Alliance of Chuxiong Prefecture, Yunnan, China, for their hard work and selfless contributions to STEMI care. In addition, we especially wish to thank Dr. Qun Mei Yao, Director of the Emergency Department, Dr. Xiao Rui Bai, Chief of the the Quality Control Office, Professor Cheng Min Yu, former Director of the Chest Pain Center of Chuxiong Prefecture, and Dr. Yuan Fei Zhang, Dr. Guo Feng Yan, Dr. Yong Ping Hu, Dr. Wen Long Hu, Dr. Lin Zhang, Dr. Yu Hu, Dr. Yun An Li, Cardiology, People’s Hospital of Chuxiong Prefecture, and Ms. Li Ying Yang, Office Secretary, Chest Pain Center of Chuxiong Prefecture, who led and contributed to the emergency rescue and quality control of STEMI care.

## Ethical issues

 The study obtained approval from the Human Research Ethics Committee, Faculty of Medicine, Prince of Songkla University, Thailand, and the Ethics Committee, People’s Hospital of Chuxiong Prefecture, Yunnan, China. Only secondary historical data were used and all patient-level data were anonymous in this study.

## Competing interests

 Authors declare that they have no competing interests.

## Authors’ contributions

 AFG and LMZ were responsible for the research design. LMZ analysed the data and wrote the initial manuscript draft, LMZ, AFG, and EBM contributed to the data interpretation and provided comments on the discussion. YPL and SCL contributed to data interpretation and the revising of the manuscript. HL, YZW, and SCW initiated the study and provided administrative support. All authors have read the manuscript and approved for submission.

## Supplementary files

Supplementary file 1 contains Figures S1- S3 and Figures S4-1 to S4-6.

Supplementary file 2 contains Tables S1- S7.
