# Supplementary material for: Health Inequalities of STEMI Care Before Implementation of a New Regional Network: A Prefecture-Level Analysis of Social Determinants of Healthcare in Yunnan, China
Source: Int J Health Policy Manag. 2021 May 11;11(8):1413–24. doi: 10.34172/ijhpm.2021.29 (PMC9808331; doi:10.34172/ijhpm.2021.29)
Supplement: Supplementary file 1 — contains Figures S1- S3 and Figures S4-1 to S4-6. [file ijhpm-11-1413-s001.pdf]

Article title: Health Inequalities of STEMI Care Before Implementation of a New Regional Network: A Prefecture-Level Analysis of Social Determinants of Healthcare in Yunnan, China

Journal name: International Journal of Health Policy and Management (IJHPM)

Authors' information: Li Mei Zhang[1,2], Alan Frederick Geater[2]*, Edward B. McNeil[2], Yun Peng Lin1, Si Chen Liu[3], Heng Luo[4,5], Yuan Zhang Wang[1,5], Shao Chang Wen[1,5]

[1]Department of Cardiology, People's Hospital of Chuxiong Prefecture, Yunnan, China.

[2]Epidemiology Unit, Faculty of Medicine, Prince of Songkla University, Hat Yai, Thailand.

[3]Faculty of Dentistry, Prince of Songkla University, Hat Yai, Thailand.

[4]People's Hospital of Chuxiong Prefecture, Yunnan, China.

[5]Executive Office, Alliance of Chuxiong Prefecture Chest Pain Centres, Yunnan, China.

(*Corresponding author: alan.g@psu.ac.th)

Supplementary file 1

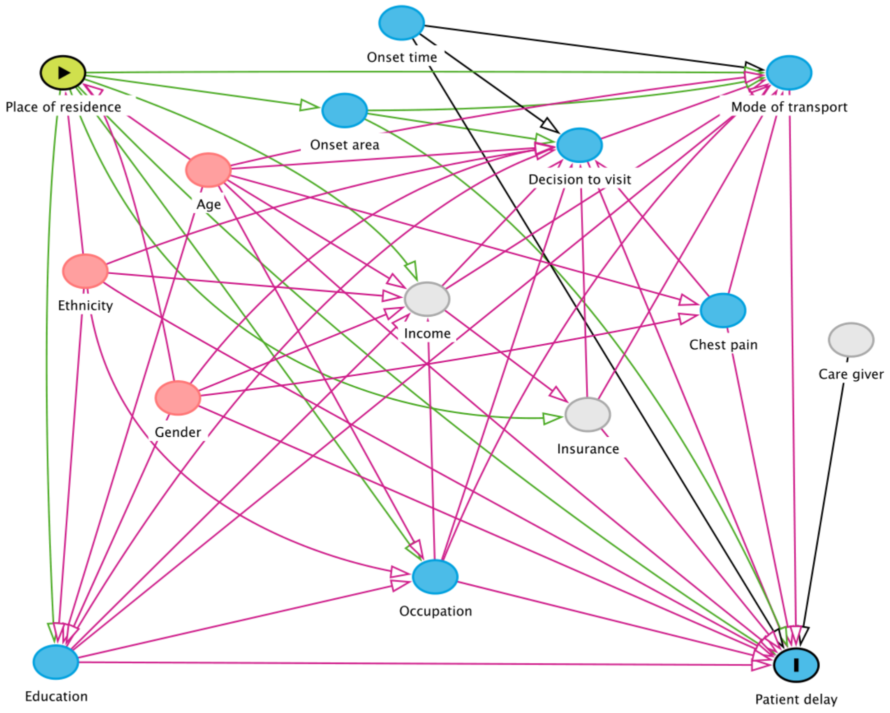

Figure S1. Example of a directed acyclic graph used to visualize causal pathways of patient delay in STEMI care

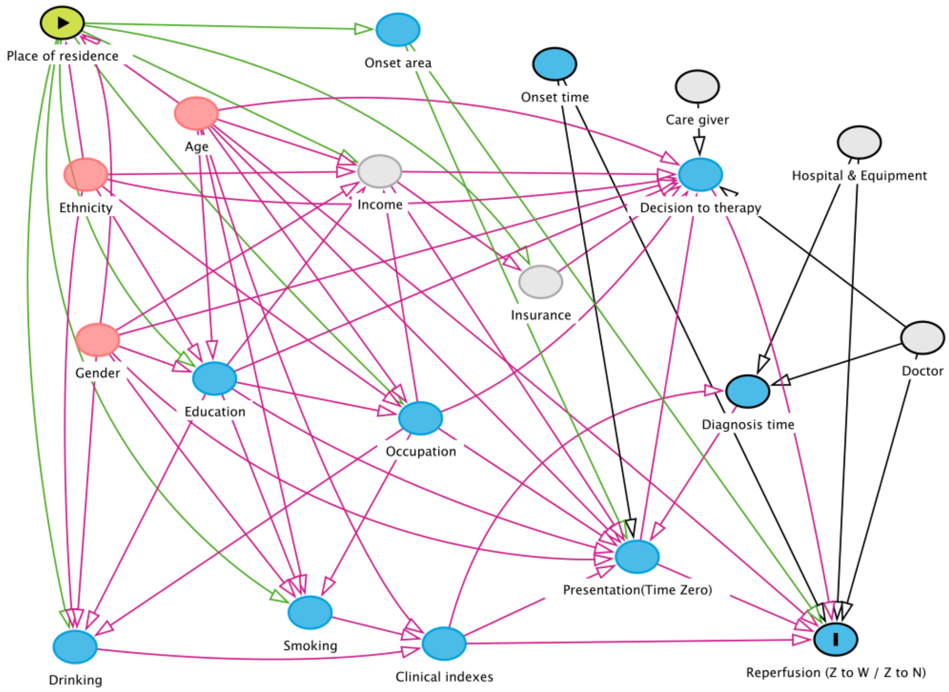

Figure S2. Example of a directed acyclic graph used to visualize causal pathways of reperfusion time in STEMI care

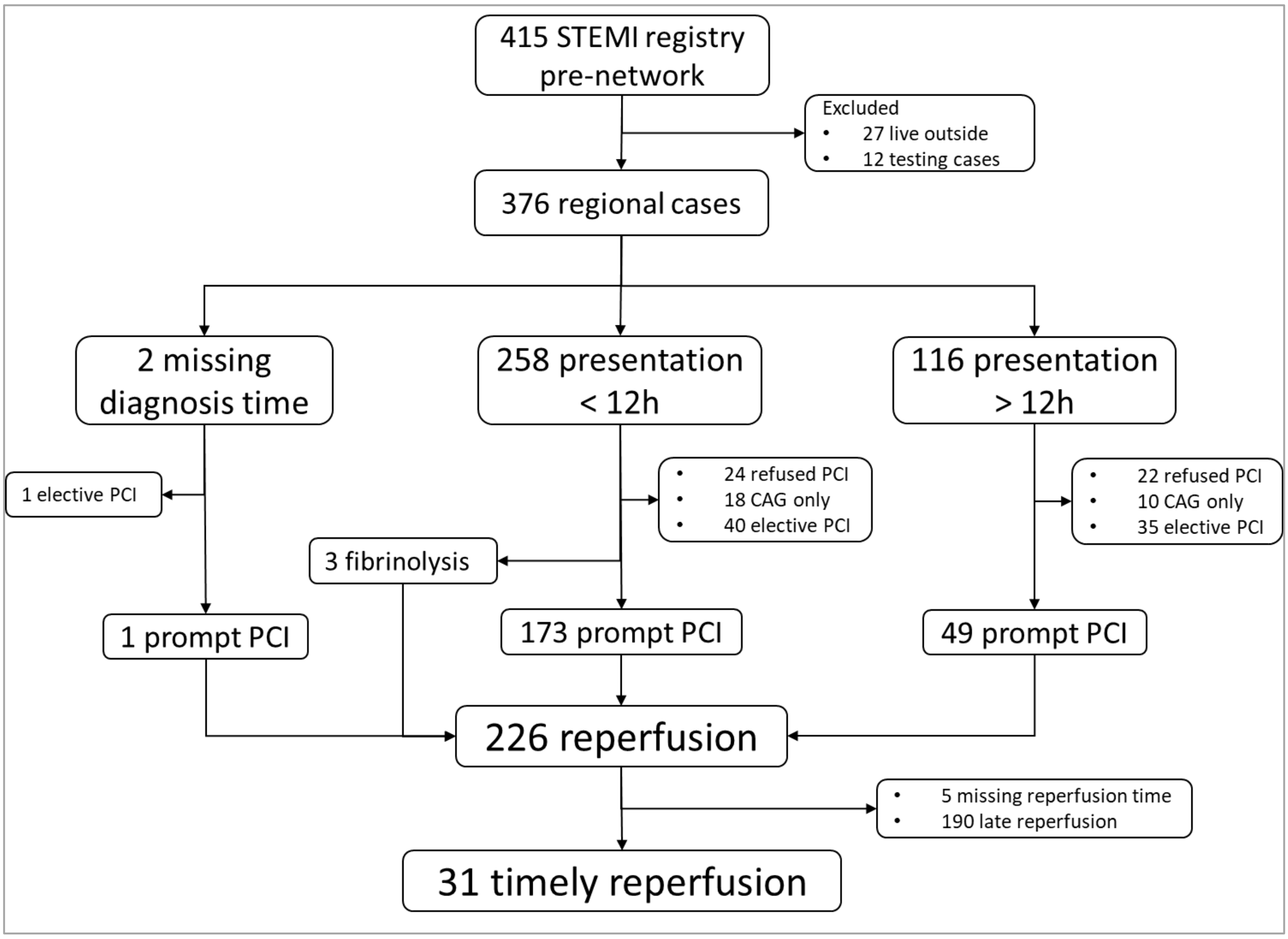

Figure S3. Diagram of STEMI care process in current study

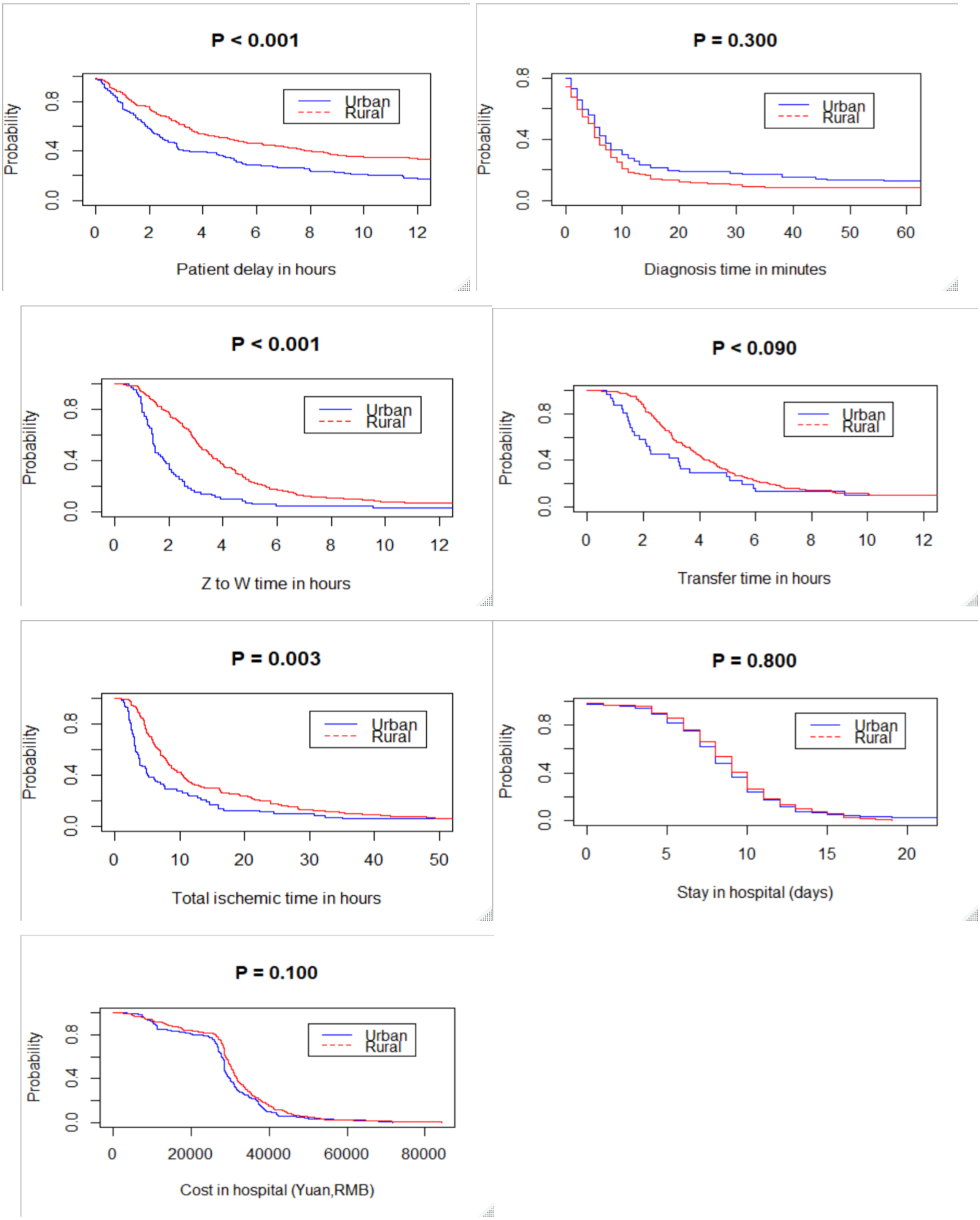

Figure S4.1. Kaplan-Meier curves of continuous outcomes of STEMI care among area groups. In Figures S4.1 to S4.6 the p-values are from the log-rank test. Z to W time is only for patients who received prompt PCI. Transfer time is only for patients contacted in a non-PCI hospital. The

Probability on the y-axis refers to the probability of having a value equal to or longer than the corresponding value on the x-axis.

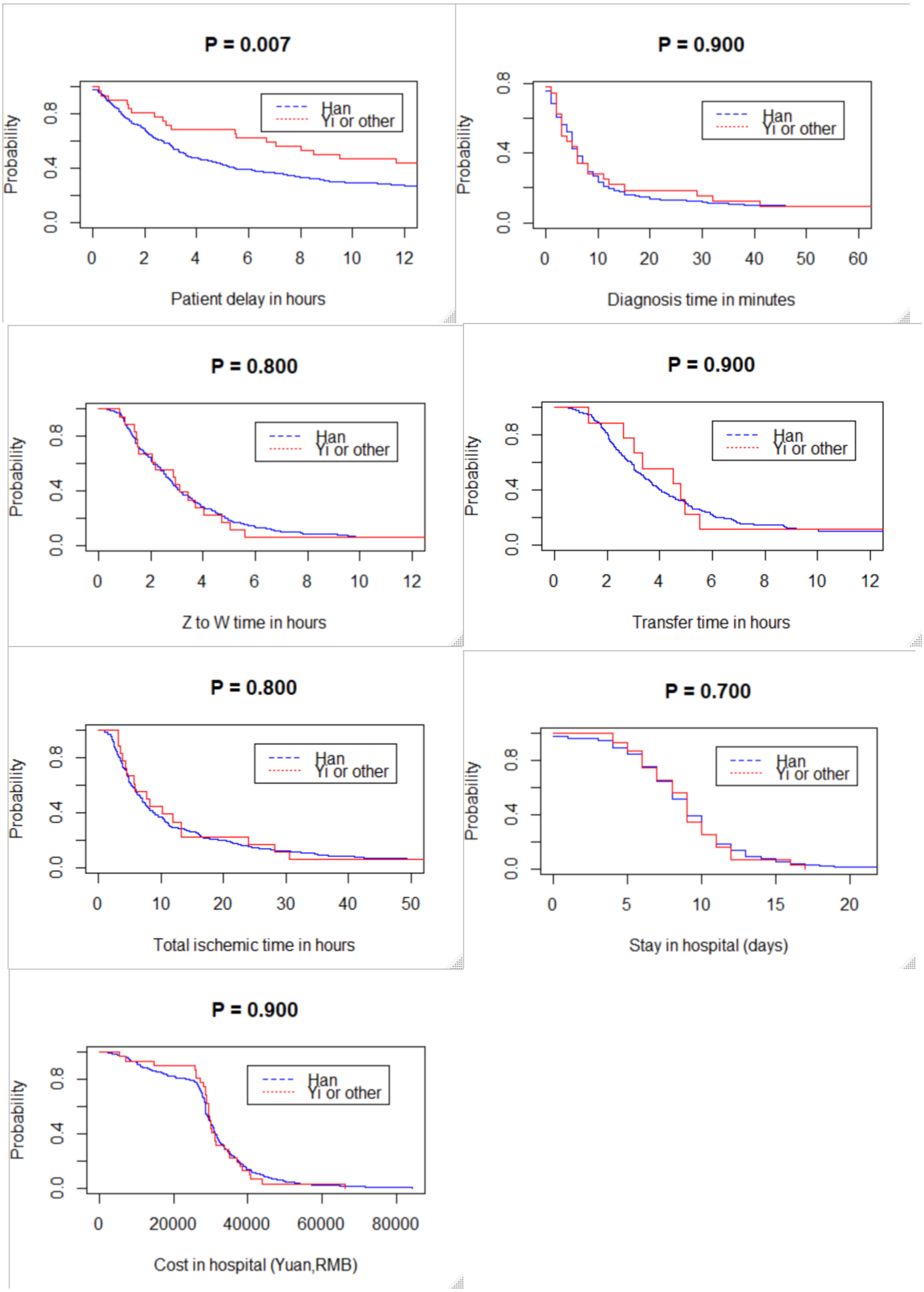

Figure S4.2. Kaplan-Meier curves of continuous outcomes of STEMI care among ethnicity groups

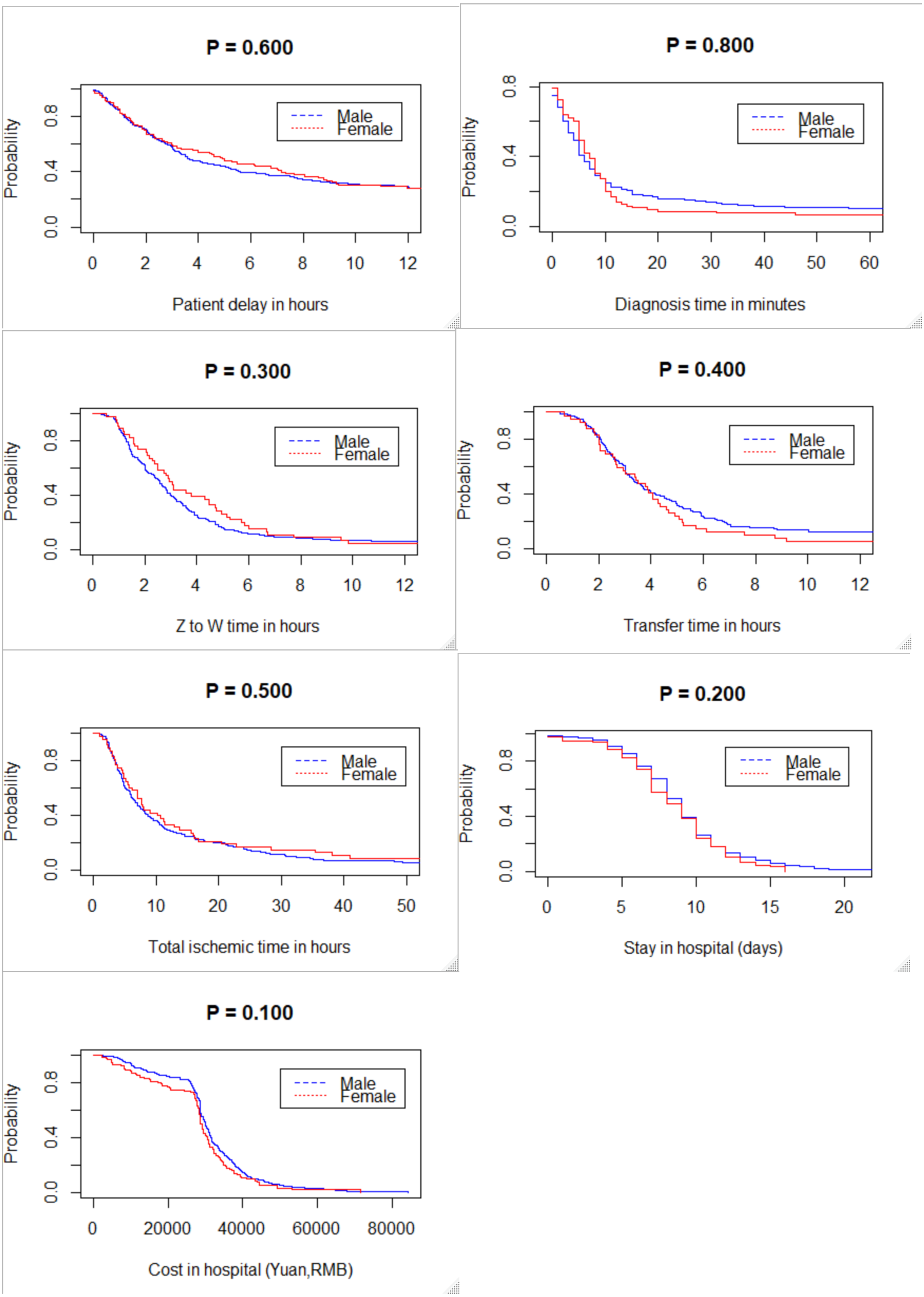

Figure S4.3. Kaplan-Meier curves of continuous outcomes of STEMI care among sex groups

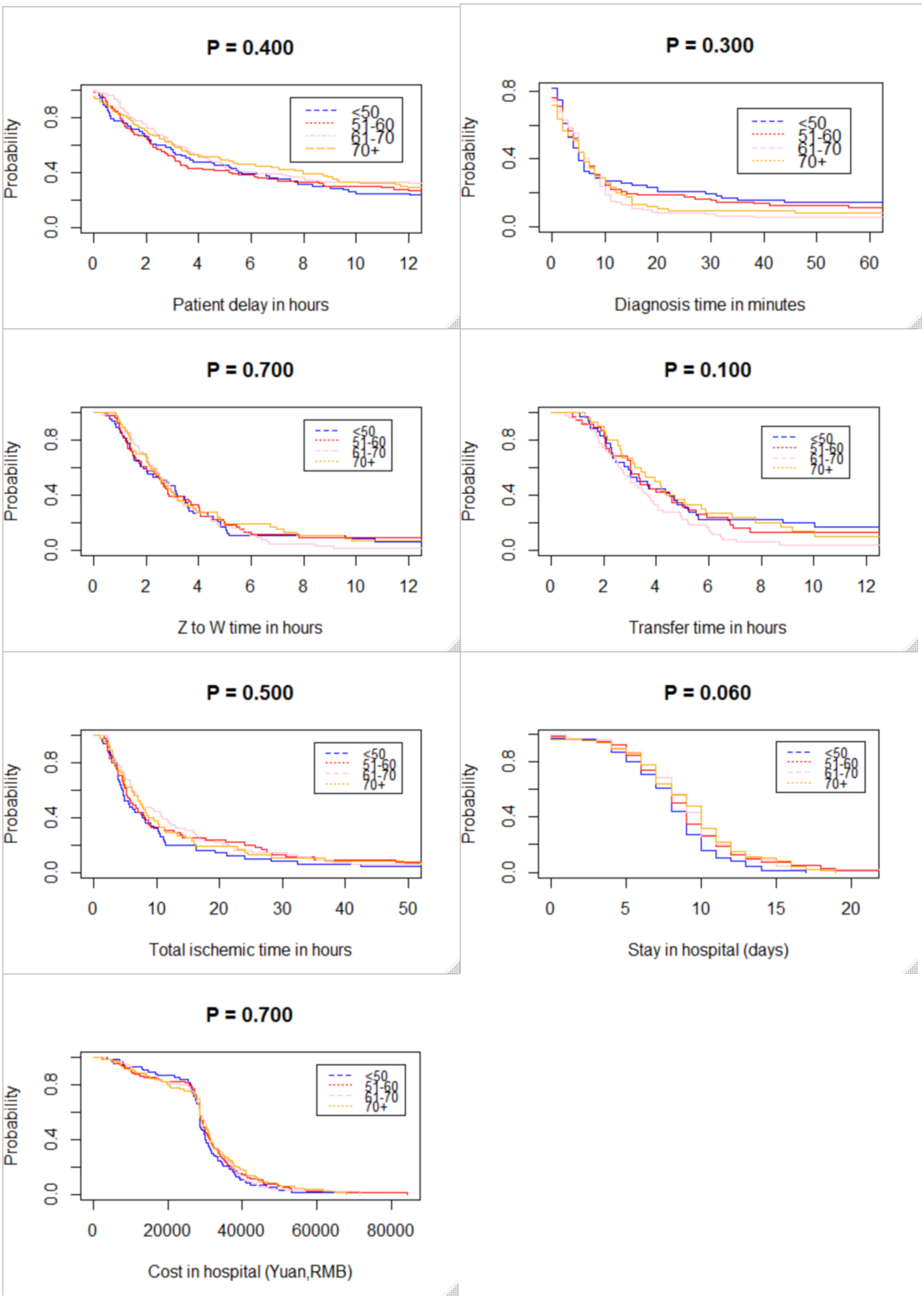

Figure S4.4. Kaplan-Meier curves of continuous outcomes of STEMI care among age groups

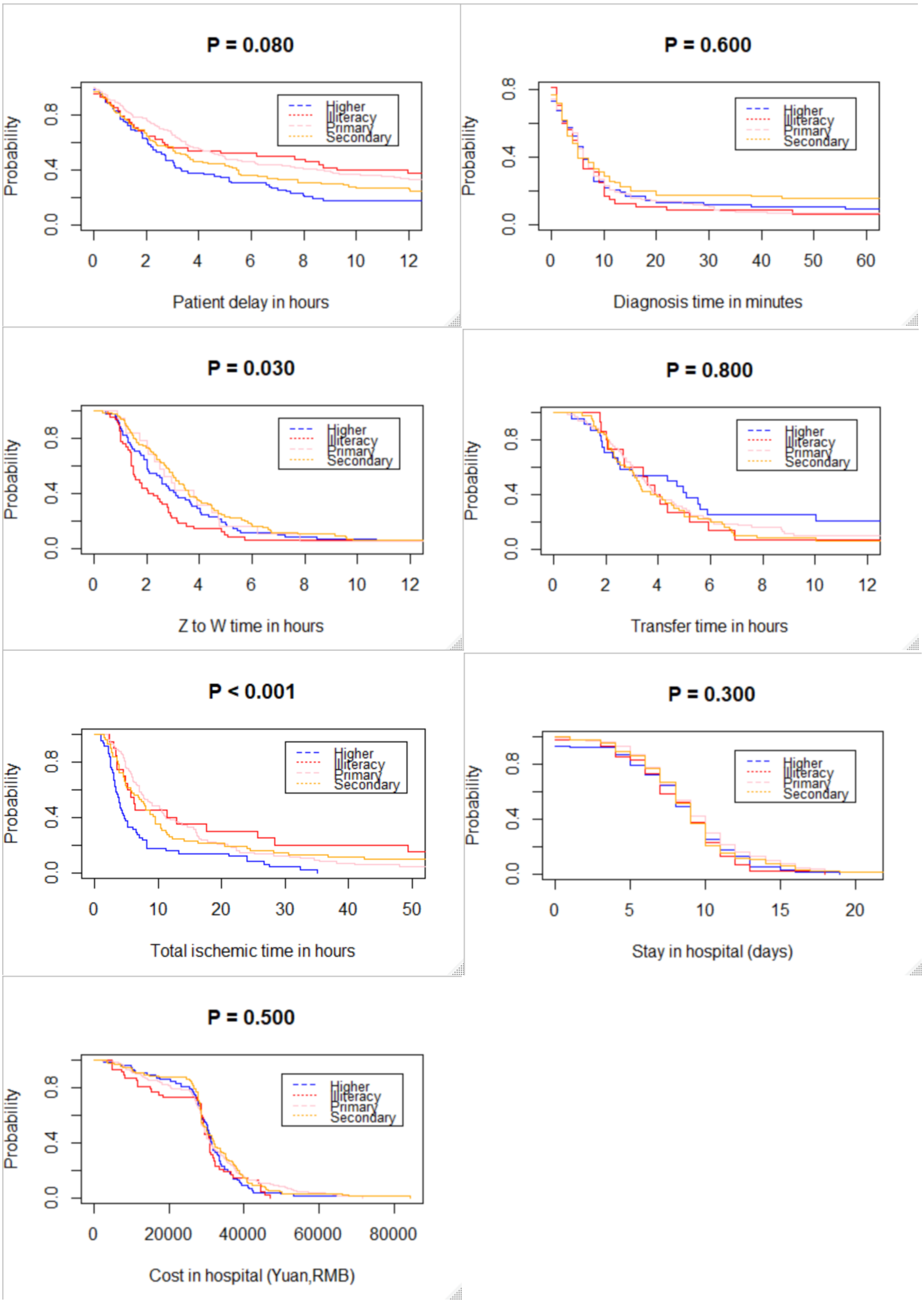

Figure S4.5. Kaplan-Meier curves of continuous outcomes of STEMI care among education groups

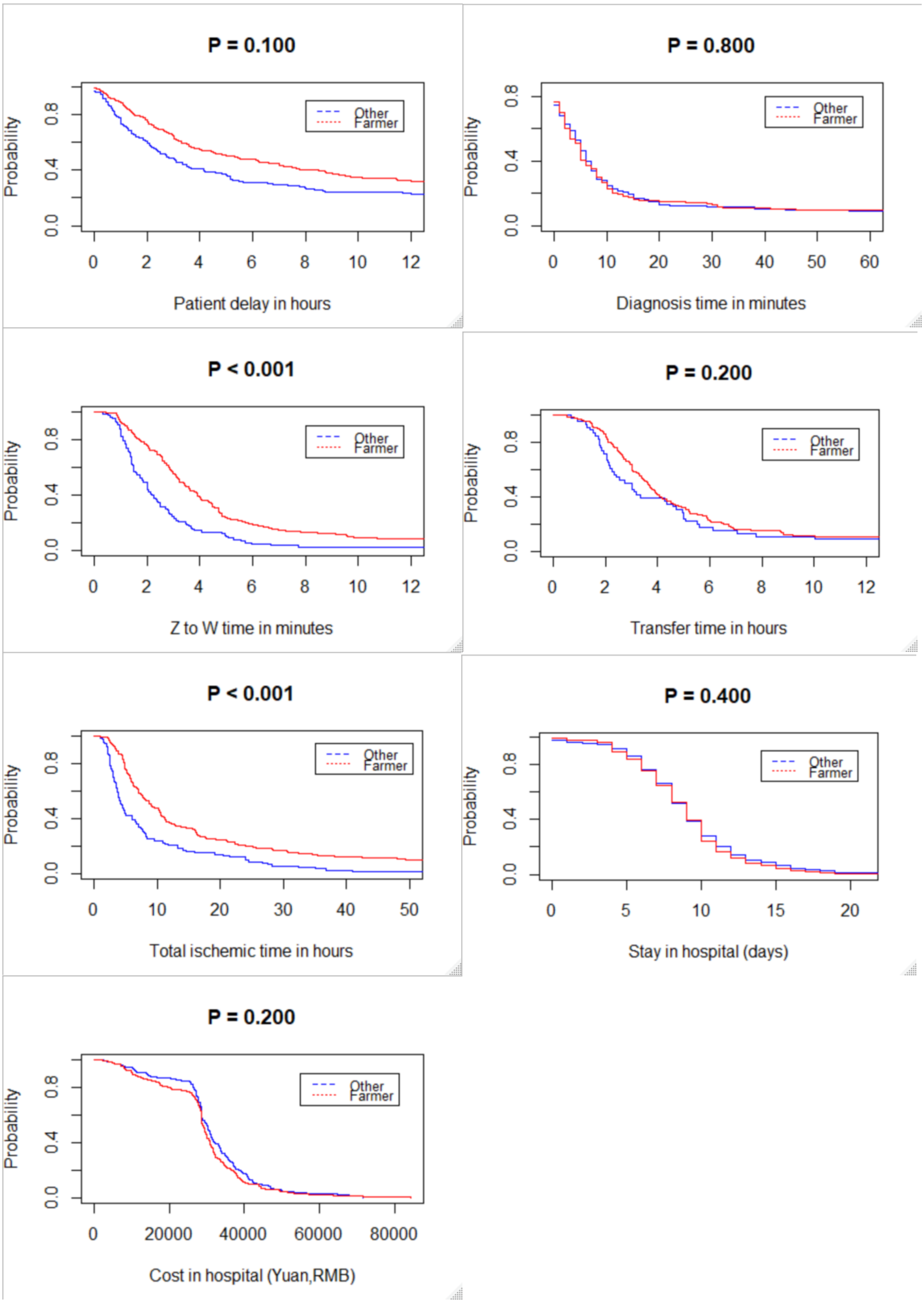

Figure S4.6. Kaplan-Meier curves of continuous outcomes of STEMI care among occupation group
