# Supplementary material for: Health Inequalities of STEMI Care Before Implementation of a New Regional Network: A Prefecture-Level Analysis of Social Determinants of Healthcare in Yunnan, China
Source: Int J Health Policy Manag. 2021 May 11;11(8):1413–24. doi: 10.34172/ijhpm.2021.29 (PMC9808331; doi:10.34172/ijhpm.2021.29)
Supplement: Supplementary file 2 — contains Tables S1- S7. [file ijhpm-11-1413-s002.pdf]

Article title: Health Inequalities of STEMI Care Before Implementation of a New Regional Network: A Prefecture-Level Analysis of Social Determinants of Healthcare in Yunnan, China

Journal name: International Journal of Health Policy and Management (IJHPM)

Authors' information: Li Mei Zhang[1,2], Alan Frederick Geater[2]*, Edward B. McNeil[2], Yun Peng Lin1, Si Chen Liu[3], Heng Luo[4,5], Yuan Zhang Wang[1,5], Shao Chang Wen[1,5]

[1]Department of Cardiology, People's Hospital of Chuxiong Prefecture, Yunnan, China.

[2]Epidemiology Unit, Faculty of Medicine, Prince of Songkla University, Hat Yai, Thailand.

[3]Faculty of Dentistry, Prince of Songkla University, Hat Yai, Thailand.

[4]People's Hospital of Chuxiong Prefecture, Yunnan, China.

[5]Executive Office, Alliance of Chuxiong Prefecture Chest Pain Centres, Yunnan, China.

(*Corresponding author: alan.g@psu.ac.th)

Supplementary file 2

Table S1. Kaplan-Meier analysis of STEMI care process (patient delay, diagnosis time and Z to W time) across social determinants of health

SDOH	N	Patient delay (hours)			Diagnosis time* (minutes)			Z to W time† (hours)		
		Events	Median (95% CI)	P-value	Events	Median (95% CI)	P	Events	Median (95% CI)	P-value
**Area of residence**				**<0.001**			0.300			**<0.001**
Urban	115	115	2.50 (1.92, 3.50)		113	5.00 (4.00, 7.00)		72	1.47 (1.38, 2.00)	
Rural	261	261	5.00 (3.67, 7.00)		261	5.00 (3.00, 5.00)		146	3.20 (2.95, 3.67)	
**Ethnicity**				**0.007**			0.900			0.800
Han	344	344	3.58 (3.08, 4.73)		342	5.00 (4.00, 5.00)		200	2.62 (2.37, 2.97)	
Other	32	32	9.01 (5.50,26.50)		32	3.50 (2.00, 8.00)		18	2.91 (1.53, 4.72)	
**Sex**				0.600			0.800			0.300
Male	280	280	3.58 (3.08, 5.00)		279	4.00 (3.00, 5.00)		172	2.58 (2.12, 2.87)	
Female	96	96	4.88 (3.00, 7.33)		95	5.00 (5.00, 8.00)		46	3.01 (2.48, 4.47)	
**Age group (yrs)**				0.400			0.300			0.700
< 50	77	77	3.65 (2.37, 6.67)		77	4.00 (3.00, 6.00)		49	2.77 (1.78, 3.57)	
51-60	97	97	3.08 (2.25, 5.50)		97	5.00 (3.00, 7.00)		54	2.58 (1.82, 3.70)	
61-70	115	115	4.35 (3.33, 6.25)		114	5.00 (4.00, 7.00)		68	2.78 (2.27, 3.22)	
> 70	87	87	4.83 (3.08, 8.75)		86	5.00 (2.00, 7.00)		47	2.67 (2.10, 3.58)	
**Education**				0.080			0.600			0.030
None	79	79	2.72 (2.07, 4.17)		78	5.00 (3.00, 7.00)		50	1.63 (1.40, 2.58)	
Primary	48	48	6.93 (2.62,15.05)		48	4.50 (2.00, 6.00)		19	2.95 (2.30, 4.75)	
Secondary	152	152	4.96 (3.67, 7.97)		151	5.00 (3.00, 6.00)		87	3.10 (2.72, 3.63)	
Tertiary	97	97	3.60 (2.37, 5.50)		97	4.00 (3.00, 7.00)		62	2.58 (2.02, 3.47)	
**Occupation**				0.100			0.800			**<0.001**
Other	152	152	2.75 (2.13, 3.82)		151	5.00 (4.00, 6.00)		93	1.83 (1.47, 2.25)	
Farmer	224	224	5.31 (3.78, 7.17)		223	5.00 (3.00, 5.00)		125	3.22 (2.90, 3.90)	

SDOH: Social determinants of health. *2 with unknown diagnosis time. †Z to W time was calculated only for patients who received prompt PCI.
P-values from the log-rank test.

Table S2. Kaplan-Meier analysis of STEMI care process (transfer time and total ischaemic time) across social determinants of health

SDOH	N	Transfer time** (hours)			Total ischaemic time‡ (hours)		
		Events	Median (95% CI)	P-value	Events	Median (95% CI)	P-value
**Area of residence**				0.090			**0.003**
Urban	115	31	2.22 (1.62, 4.95)		73	3.83 (3.23, 5.97)	
Rural	261	128	3.66 (3.07, 4.25)		149	8.17 (7.07,10.17)	
**Ethnicity**				0.900			0.800
Han	344	150	3.38 (3.00, 3.95)		204	6.97 (5.97, 8.17)	
Other	32	9	4.52 (1.28, 5.50)		18	7.97 (4.50,24.05)	
**Sex**				0.400			0.500
Male	280	117	3.33 (3.00, 4.17)		174	6.69 (5.83, 8.17)	
Female	96	42	3.45 (2.63, 4.37)		48	7.72 (5.63,11.47)	
**Age group (yrs)**				0.100			0.500
< 50	77	36	3.46 (2.47, 5.07)		50	5.58 (4.32, 9.50)	
51-60	97	38	3.38 (3.00, 5.20)		55	6.25 (5.13, 8.78)	
61-70	115	55	3.03 (2.58, 3.87)		69	7.60 (6.05,11.92)	
> 70	87	30	4.04 (3.08, 5.87)		48	7.49 (6.03,10.32)	
**Education**				0.800			**<0.001**
None	79	24	4.52 (2.47, 5.87)		51	3.92 (3.25, 5.20)	
Primary	48	15	3.58 (2.63, 5.92)		20	6.29 (4.92,28.32)	
Secondary	152	70	3.48 (3.00, 4.17)		89	8.98 (7.08,11.92)	
Tertiary	97	50	3.21 (2.53, 4.50)		62	7.78 (5.63, 9.62)	
**Occupation**				0.200			**<0.001**
Other	152	46	2.87 (2.17, 4.58)		94	4.38 (3.75, 6.52)	
Farmer	224	113	3.63 (3.07, 4.08)		128	8.99 (7.33,11.20)	

SDOH: Social determinants of health. **Transfer time calculated only for patients who were treated in a non-PCI hospital. P-values from log-rank test.

Table S3. Kaplan-Meier analysis of STEMI care process (length of stay in hospital and hospital cost) across social determinants of health

SDOH	N	Length of stay in-hospital (days)			Hospital cost (yuan)		
		Events	Median (95% CI)	P-value	Events	Median (95% CI)	P-value
**Area of residence**				0.800			0.100
Urban	115	115	8.00 (8.00, 9.00)		115	28402 (25814, 34054)	
Rural	261	261	9.00 (8.00, 9.00)		261	30338 (27337, 35605)	
**Ethnicity**				0.700			0.900
Han	344	344	9.00 (8.00, 9.00)		344	29550 (26685, 35402)	
Other	32	32	9.00 (8.00,10.00)		32	29836 (28300, 34938)	
**Sex**				0.200			0.100
Male	280	280	9.00 (8.00, 9.00)		280	29908 (26977, 36140)	
Female	96	96	8.00 (7.00, 9.00)		96	28812 (23412, 33748)	
**Age group (yrs)**				0.060			0.700
< 50	77	77	8.00 (8.00, 9.00)		77	28489 (26557, 33439)	
51-60	97	97	9.00 (8.00, 9.00)		97	29519 (27028, 35002)	
61-70	115	115	9.00 (8.00,10.00)		115	30334 (26837, 35532)	
> 70	87	87	9.00 (8.00,10.00)		87	29502 (26631, 36443)	
**Education**				0.300			0.500
None	79	79	8.00 (8.00, 9.00)		79	30338 (26913, 33616)	
Primary	48	48	9.00 (7.00,10.00)		48	29438 (18170, 32179)	
Secondary	152	152	9.00 (8.00,10.00)		152	29359 (26286, 35768)	
Higher	97	97	9.00 (8.00, 9.00)		97	29902 (27620, 37143)	
**Occupation**				0.400			0.200
Other	152	152	9.00 (8.00, 9.00)		152	30336 (27169, 36850)	
Farmer	224	224	9.00 (8.00, 9.00)		224	29374 (26194, 34430)	

SDOH: Social determinants of health. P-values from log-rank test.

Table S4. Cox regression of STEMI care process (patient delay, diagnosis time and Z to W time) across social determinants of health

SDOH	Patient delay			Diagnosis time			Z to W time[†]			Minimal adjustment set
	Adj. HR (95% CI)	P (Wald)	P (LR)	Adj. HR (95% CI)	P (Wald)	P (LR)	Adj. HR (95% CI)	P (Wald)	P (LR)	
**Area (Ref. Urban)**						0.228			**< 0.001**	Age, ethnicity, sex.
Rural	0.65 (0.52,0.82)	< 0.001	**< 0.001**	1.15 (0.92,1.44)	0.232		0.42 (0.31,0.57)	< 0.001		
**Ethnicity (Ref. Han)**						0.946			0.813	No adjustment is necessary.
Other	0.60 (0.41,0.87)	0.007	**0.004**	1.01 (0.70,1.46)	0.946		1.06 (0.65,1.72)	0.812		
**Sex (Ref. Male)**						0.815			0.242	No adjustment is necessary.
Female	0.94 (0.75,1.19)	0.625	0.623	1.03 (0.81,1.30)	0.814		0.83 (0.60,1.14)	0.251		
**Age (Ref. <50 yrs)**			0.360			0.349			0.702	No adjustment is necessary.
51-60	0.97 (0.72,1.31)	0.858		0.95 (0.70,1.29)	0.749		0.87 (0.59,1.29)	0.496		
61-70	0.80 (0.59,1.06)	0.123		1.19 (0.89,1.59)	0.253		1.01 (0.70,1.46)	0.965		
> 70	0.88 (0.65,1.19)	0.409		1.16 (0.85,1.58)	0.351		0.83 (0.56,1.25)	0.379		
**Education (Ref. Higher)**			0.305			0.780			0.058	Area, ethnicity, sex, age.
None	1.20 (0.89,1.62)	0.239		1.13 (0.84,1.53)	0.423		1.40 (0.96,2.05)	0.085		
Primary	0.85 (0.57,1.26)	0.411		1.14 (0.77,1.69)	0.505		1.09 (0.60,1.96)	0.784		
Secondary	0.93 (0.71,1.22)	0.608		1.14 (0.87,1.49)	0.346		0.82 (0.57,1.18)	0.292		
**Occupation (Ref. Other)**						0.959			**< 0.001**	Area, age, ethnicity, sex, education.
Farmer	0.88 (0.69,1.13)	0.318	0.319	0.99 (0.78,1.27)	0.959		0.55 (0.39,0.78)	< 0.001		

SDOH: Social determinants of health. [†]Z to W time: only for patients who received prompt PCI. HR: Hazard ratio. LR: Likelihood ratio test.

Table S5. Cox regression of STEMI care process (transfer time and total ischaemic time) across social determinants of health

SDOH	Transfer time*			Total ischaemic time**			Minimal adjustment set
	Adj. HR (95% CI)	P (Wald)	P (LR)	Adj. HR (95% CI)	P (Wald)	P (LR)	
**Area (Ref. Urban)**			0.149			**0.005**	Age, ethnicity, sex.
Rural	0.73 (0.49,1.10)	0.138		0.66 (0.50,0.88)	0.004		
**Ethnicity (Ref. Han)**			0.877			0.800	No adjustment is necessary.
Other	0.95 (0.48,1.86)	0.878		0.94 (0.58,1.52)	0.802		
**Sex (Ref. Male)**			0.364			0.473	No adjustment is necessary.
Female	1.18 (0.83,1.69)	0.358		0.89 (0.65,1.23)	0.478		
**Age (Ref. <50 yrs)**			0.145			0.476	No adjustment is necessary.
51-60	0.91 (0.57,1.45)	0.701		0.83 (0.56,1.22)	0.339		
61-70	1.36 (0.89,2.08)	0.159		0.74 (0.52,1.07)	0.115		
> 70	0.85 (0.52,1.38)	0.503		0.87 (0.58,1.29)	0.483		
**Education (Ref. Higher)**			0.701			**0.008**	Area, ethnicity, sex, age.
None	0.80 (0.48,1.34)	0.393		1.80 (1.23,2.64)	0.003		
Primary	0.84 (0.44,1.60)	0.591		0.94 (0.53,1.65)	0.821		
Secondary	0.79 (0.53,1.19)	0.261		0.93 (0.65,1.35)	0.716		
**Occupation (Ref. Other)**			**0.045**			**0.013**	Area, ethnicity, sex, age, education.
Farmer	0.62 (0.40,0.98)	0.040		0.64 (0.45,0.90)	0.012		

SDOH: Social determinants of health. *Transfer time: only for patients who contacted non-PCI hospital. **Total ischaemic time: only for patients who received prompt reperfusion therapy. HR: Hazard ratio. LR: Likelihood ratio test.

Table S6. Cox regression of STEMI care process (length of stay in hospital and hospital cost) across social determinants of health

SDOH	Length of stay in hospital			Hospital cost			Minimal adjustment set
	**Adj. HR (95% CI)**	**P (Wald)**	**P (LR)**	**Adj. HR (95% CI)**	**P (Wald)**	**P (LR)**	
**Area(Ref.Urban)**			0.756			0.053	Age, ethnicity, sex.
Rural	0.97 (0.77,1.21)	0.755		0.80 (0.64,1.00)	0.050		
**Ethnicity (Ref.Han)**			0.691			0.868	No adjustment is necessary.
Other	1.08 (0.75,1.55)	0.688		1.03 (0.72,1.48)	0.868		
**Sex (Ref.Male)**			0.196			0.151	No adjustment is necessary.
Female	1.17 (0.93,1.48)	0.191		1.19 (0.94,1.50)	0.145		
**Age (Ref.<50 years)**			0.071			0.702	No adjustment is necessary.
51-60 years	0.74 (0.55,1.00)	0.049		0.86 (0.64,1.16)	0.333		
61-70 years	0.71 (0.53,0.95)	0.020		0.88 (0.66,1.18)	0.402		
> 70 years	0.68 (0.50,0.92)	0.013		0.84 (0.62,1.14)	0.262		
**Education (Ref.Higher)**			0.512			0.544	Area, ethnicity, sex, age.
None	1.09 (0.81,1.48)	0.562		1.09 (0.81,1.48)	0.569		
Primary	1.20 (0.79,1.81)	0.396		1.29 (0.87,1.93)	0.210		
Secondary	0.94 (0.71,1.26)	0.696		1.02 (0.77,1.34)	0.909		
**Occupation (Ref.Other)**			0.407			0.057	Area, ethnicity, sex, age, education.
Farmer	1.11 (0.87,1.41)	0.408		1.27 (0.99,1.62)	0.059		

SDOH: Social determinants of health. # Trend over age groups for hospital stay HR=0.89 [0.81, 0.98], P=0.022

Table S7. Time-varying effect models between rural and urban residents

	Z to W time*					
	HR	95% CI lower	95% CI upper	p (Z-test)	p (LR-test)	p (PH)
**Area of residence (Ref. Urban)**					**<.001**	.498
Rural * < 3 hour	0.29	0.20	0.42	<.001		.514
Rural * >=3 hour	0.89	0.25	3.12	.851		.714
	**Total ischemic time†**					
	HR	95% CI lower	95% CI upper	p (Z-test)	p (LR-test)	p (PH)
**Area of residence (Ref. Urban)**					**<.001**	.581
Rural * < 6 hour	0.37	0.25	0.56	<.001		.098
Rural * >=6 hour	1.16	0.75	1.80	.502		.962

* For patients who received prompt PCI.
